# Effects of ABCB1 gene polymorphism on the efficacy of antidepressant drugs: A protocol for systematic review and meta-analysis

**DOI:** 10.1097/MD.0000000000026411

**Published:** 2021-07-16

**Authors:** Xiaoying Zheng, Zejuan Fu, Xiaomei Chen, Mingxia Wang, Rixia Zhu

**Affiliations:** a Cross Infection Control Office; b Department of Nursing; c Operating Room, Second Affiliated Hospital of Hainan Medical College; d Department of Neurology and Geriatrics of Medicine, Hainan Province Anning Hospital, Haikou, Hainan Province, China.

**Keywords:** ABCB1, antidepressant drugs, meta-analysis, polymorphism, protocol

## Abstract

**Background:**

Antidepressant drugs are mainly used to treat depression clinically. ABCB1 affects the P-glycoprotein activity and changes the amount of drugs in the blood tissue barrier that can be squeezed back into the blood, thus affecting the efficacy of antidepressants. In this present study, Meta-analysis was performed to further investigate the influences of ABCB1 gene polymorphism on antidepressant response.

**Methods:**

Relevant literatures were searched from the PubMed, EMBASE, Web of Science, Chinese National Knowledge Infrastructure, Chinese Science and Technique Journals Database, China Biology Medicine disc, and Wan Fang databases up to May 2021 without any language restrictions. STATA 16.0 software was applied for this meta-analysis. Odds ratio (OR) and its corresponding 95% confidence interval (CI) were calculated.

**Results:**

The results of this meta-analysis will be submitted to a peer-reviewed journal for publication.

**Conclusion:**

This meta-analysis will summarize the effects of ABCB1 gene polymorphism on antidepressant response.

## 1 Introduction

Depression is a kind of common emotional disorder, characterized by significant and persistent somatic symptoms such as low mood, decreased activity ability, slow thinking, and cognitive function.^[1]^ Although the etiology and pathogenesis of depression are still unclear at present, a large number of studies have confirmed that genetic and environmental factors are important in the pathogenesis of depression.^[2,3]^

At present, a wide variety of drugs and methods can be adopted to treat depression. However, in clinical practices, it has been discovered that even with standard doses of antidepressants for 6 to 8 weeks, 35% to 45% of patients do not fully recover to their pre-onset state.^[4,5]^ Moreover, the clinical effects of drugs are often delayed by 2 to 4 weeks. Meanwhile, 12% to 15% of patients cannot tolerate the adverse reactions of the drug and discontinued treatment.^[6,7]^ Many studies have identified that genetic variations may partially explain individual differences in response to antidepressants.^[8]^

A large number of studies have revealed that P-glycoprotein is involved in the transmembrane transport of many antidepressants.^[9–11]^ Many antidepressants act as substrates for P-glycoprotein.^[12]^ ABCB1 gene is located on human chromosome 7. On the other hand, as an important component of the blood-brain barrier and gastrointestinal barrier, its encoded 1280 amino acid transporter P-glycoprotein can limit drug infiltration and accumulation in the brain, and regulate the effectiveness and toxicity of drugs.^[13–15]^ Therefore, ABCB1 gene polymorphism may affect the function of P-glycoprotein, thus changing the concentration of substrate drugs in the brain, with various degrees of impacts on the clinical efficacy of anti-depression drugs.^[16]^ According to the existing research results, ABCB1 gene polymorphism has a certain correlation with the efficacy of antidepressants.

It is of great significance to improve the response rate of antidepressants from the perspective of genetics. At present, the relationship between ABCB1 gene polymorphism and the efficacy of antidepressants is still controversial.^[8,17–20]^ To date, no meta-analysis has been carried out on the relationship between ABCB1 gene polymorphism and antidepressant response. Therefore, we conducted a meta-analysis to elucidate the association between ABCB1 gene polymorphism and antidepressant efficacy.

## 2 Methods

### 2.1 Study registration

The protocol of this review was registered in OSF (OSF registration number: DOI 10.17605/OSF.IO/R28W7). It was reported to follow the statement guidelines of preferred reporting items for systematic reviews and meta-analyses protocol.^[21]^

### 2.2 Inclusion criteria

(1) Published studies on the effects of ABCB1 gene polymorphisms on the efficacy of antidepressants;
(2) The study participants were depressed patients;
(3) The relationship between ABCB1 gene polymorphism and antidepressants can be obtained from original literatures;
(4) The antidepressants all patients took were the substrates of P-glycoprotein.

### 2.3 Exclusion criteria

The exclusion criteria included case reports, meta-analysis, review articles, and studies without detailed genotype data.

### 2.4 Search strategy

Relevant studies in PubMed, EMBASE, Web of Science, Chinese National Knowledge Infrastructure, Chinese Science and Technique Journals Database, China Biology Medicine disc, and Wan Fang databases were searched by May 2021. The search strategy was based on the following key words: “antidepressant”; “response”; “ABCB1”; “genetic polymorphisms”; and others. The search strategy for PubMed is displayed in Table 1.

**Table 1:** Search strategy in PubMed database.

Number	Search terms
#1	Antidepressive Agents [MeSH]
#2	Antidepressants[Title/Abstract]
#3	Thymoanaleptics[Title/Abstract]
#4	Thymoleptics[Title/Abstract]
#5	Antidepressant Drugs[Title/Abstract]
#6	Agents, Antidepressive[Title/Abstract]
#7	Drugs, Antidepressant[Title/Abstract]
#8	or/1–7
#9	ABCB1[Title/Abstract]
#10	MDR-1[Title/Abstract]
#11	or/9–10
#12	Variation[Title/Abstract]
#13	Mutation[Title/Abstract]
#14	Polymorph∗[Title/Abstract]
#15	Variants[Title/Abstract]
#16	Variant[Title/Abstract]
#17	Susceptibility[Title/Abstract]
#18	or/12–17
#19	#8 and #11 and #18

### 2.5 Data collection and analysis

#### 2.5.1 Selection of studies

**Figure 1:**
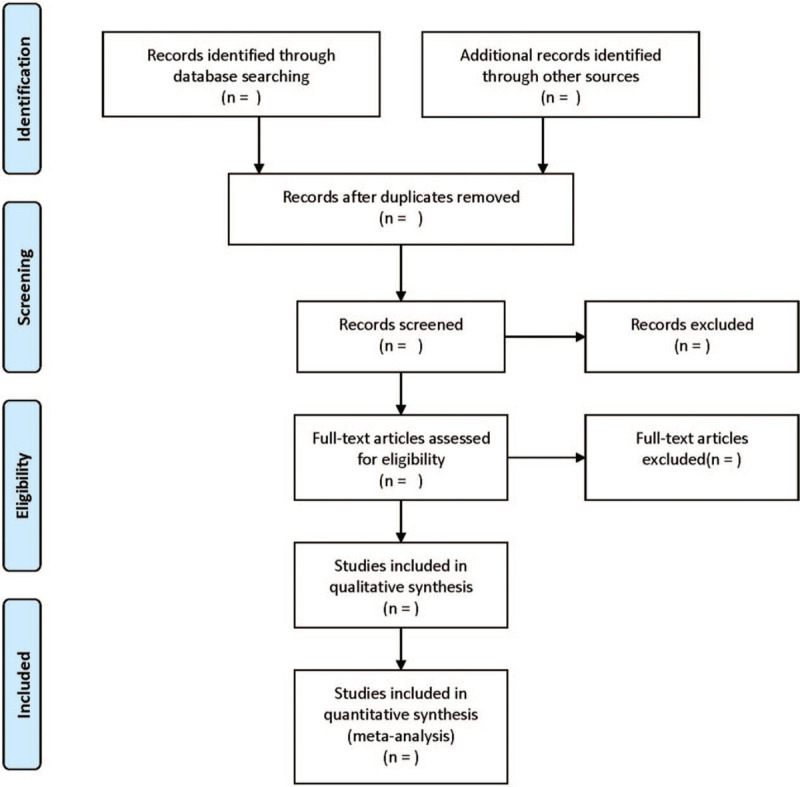
Flow diagram of study selection process.

The flowchart is demonstrated in Fig. 1. According to the inclusion criteria, 2 researchers independently read the literature and extracted the data. In case of disagreement, a third researcher will discuss and negotiate.

#### 2.5.2 Data extraction

Data extraction was carried out for all literatures that were included in the final analysis. The extracted contents include first author, year of publication, ethnicity of the case studied, year, age, number of people, scale used in the study, type of antidepressant used by patients, number of days of treatment, ABCB1 sites studied, and so on.

#### 2.5.3 Methodology quality assessment

We investigated the quality of each study based on the 9-point Newcastle-Ottawa Scale.^[22]^ If the Newcastle-Ottawa Scale score of the literature is ≥6, it can be considered as high quality.^[23]^

#### 2.5.4 Dealing with missing data

If there exists insufficient or missing data in the literature, we would only analyze the currently available data and discuss its potential value.

#### 2.5.5 Statistical analysis

The combined odds ratio (OR) and 95% confidence interval (CI) were used to evaluate the effects of ABCB1 polymorphism on antidepressant efficacy. Five genotypic models were adopted to detect the relationship between SNPs and response rate: allele model (T vs C), heterozygote model (TC vs CC), homozygote model (TT vs CC), dominant model (TT + TC vs CC), and recessive model (TT vs TC + CC). Heterogeneity was tested by *Q* statistic and quantified by *I*^2^ value. If *P* > .1or *I*^2^ <50%, the fixed effect model was used for analysis; if *P* < .1 or *I*^2^ > 50%, it indicated the existence of large heterogeneity, and the random effects model analysis should be used. All analyses were carried out with STATA 16.0 (STATA Corporation, College Station, TX).

#### 2.5.6 Subgroup analysis

We performed subgroup analyses by ethnicity, type of drug, and duration of treatment.

#### 2.5.7 Sensitivity analysis

The eligible study was sequentially removed to perform the sensitivity analysis.

#### 2.5.8 Assessment of publication biases

If no <10 studies are included, funnel charts are used to assess publication bias.^[24,25]^

#### 2.5.9 Ethics and dissemination

The content of this article does not involve moral approval or ethical review and would be presented in print or at relevant conferences.

## 3 Discussion

Being known as multidrug resistance gene, ABCB1 gene is located at 7q21 and encodes p-glycoprotein.^[26]^ Its main function include the prevention of drugs and foreign substances from entering body tissues, such as antidepressants, anti-tumor drugs, glucocorticoids, and amyloid proteins.^[27–29]^ Due to the exogenous effects of P-glycoprotein on exogenous substances and drugs, ABCB1 gene polymorphism and different P-glycoprotein expression may lead to different populations or individuals with different susceptibility to some diseases.^[14]^ Previous reports on the relationship between ABCB1 gene mutation and antidepressant efficacy are inconsistent. Through meta-analysis, this study further explored the relationship between ABCB1 gene polymorphism and the efficacy of antidepressants, so as to provide an etiological basis for individualized treatment in patients suffering from depression.

## Author contributions

**Conceptualization:** Rixia Zhu, Xiaoying Zheng.

**Data curation:** Rixia Zhu, Zejuan Fu.

**Formal analysis:** Zejuan Fu, Xiaomei Chen.

**Funding acquisition:** Rixia Zhu.

**Investigation:** Zejuan Fu.

**Methodology:** Mingxia Wang.

**Project administration:** Rixia Zhu.

**Resources:** Xiaomei Chen.

**Software:** Xiaomei Chen.

**Supervision:** Rixia Zhu.

**Validation:** Mingxia Wang, Xiaoying Zheng.

**Visualization:** Mingxia Wang.

**Writing – original draft:** Rixia Zhu, Xiaoying Zheng.

**Writing – review & editing:** Rixia Zhu, Xiaoying Zheng.
